# Medical cannabis for the reduction of opioid dosage in the treatment of non-cancer chronic pain: a systematic review

**DOI:** 10.1186/s13643-020-01425-3

**Published:** 2020-07-28

**Authors:** Babasola O. Okusanya, Ibitola O. Asaolu, John E. Ehiri, Linda Jepkoech Kimaru, Abidemi Okechukwu, Cecilia Rosales

**Affiliations:** 1 Department of Health Promotion Sciences, Mel and Enid Zuckerman College of Public Health, University of Arizona, Tucson, AZ USA; 2 Department of Health Behavior and Health Promotion, Mel and Enid Zuckerman College of Public Health, University of Arizona, Tucson, USA; 3 Division of Public Health Practice & Translational Research, Mel and Enid Zuckerman College of Public Health, University of Arizona, 714 E. Van Buren Street, Suite 119, UA Phoenix Plaza Building 4, Phoenix, AZ 85006 USA

**Keywords:** Opioid epidemic, Medical cannabis, Opioid substitution, Opioid crisis

## Abstract

**Background:**

Medical cannabis (MC) is currently being used as an adjunct to opiates given its analgesic effects and potential to reduce opiate addiction. This review assessed if MC used in combination with opioids to treat non-cancer chronic pain would reduce opioid dosage.

**Methods:**

Four databases—Ovid (Medline), Psyc-INFO, PubMed, Web of Science, and grey literature—were searched to identify original research that assessed the effects of MC on non-cancer chronic pain in humans. Study eligibility included randomized controlled trials, controlled before-and-after studies, cohort studies, cross-sectional studies, and case reports. All databases were searched for articles published from inception to October 31, 2019. Cochrane’s ROBINS-I tool and the AXIS tool were used for risk of bias assessment. PRISMA guidelines were followed in reporting the systematic review.

**Results:**

Nine studies involving 7222 participants were included. There was a 64–75% reduction in opioid dosage when used in combination with MC. Use of MC for opioid substitution was reported by 32–59.3% of patients with non-cancer chronic pain. One study reported a slight decrease in mean hospital admissions in the past calendar year (*P* = .53) and decreased mean emergency department visits in the past calendar year (*P* = .39) for patients who received MC as an adjunct to opioids in the treatment of non-cancer chronic pain compared to those who did not receive MC. All included studies had high risk of bias, which was mainly due to their methods.

**Conclusions:**

While this review indicated the likelihood of reducing opioid dosage when used in combination with MC, we cannot make a causal inference. Although medical cannabis’ recognized analgesic properties make it a viable option to achieve opioid dosage reduction, the evidence from this review cannot be relied upon to promote MC as an adjunct to opioids in treating non-cancer chronic pain. More so, the optimal MC dosage to achieve opioid dosage reduction remains unknown. Therefore, more research is needed to elucidate whether MC used in combination with opioids in the treatment of non-cancer chronic pain is associated with health consequences that are yet unknown.

**Systematic review registration:**

This systematic review was not registered.

## Background

Pain is an unpleasant experience that is subjective in nature; it differs in duration and etiology. Chronic pain, often described as pain that persists for a minimum of 3 months, may stem from an initial injury (e.g., back sprain), illness, or an unexplained cause [1]. Non-cancer chronic pain differs from cancer pain because cancer pain arises from the invasion of a tumor and the interaction among tumor cells, the nervous system, and an individual’s immune system [2, 3]. Cancer pain often advances as the disease progresses [2]. Because of differences in etiology and management of these forms of pain, this review focused on non-cancer chronic pain.

Figures from the 2016 National Health Interview Survey estimate that one in five (20.4%; 50 million) Americans suffer from non-cancer chronic pain [2]. The burden of chronic pain among Americans is higher among the following demographics: (1) females (22.1%) versus males (18.6%), (2) non-Hispanic White (23.0%) versus other races/ethnicities, and (3) adults 45 years or older [2]. The magnitude of non-cancer chronic pain has led to the proliferation of opioid prescriptions and addiction which is currently a public health concern in the USA [4]. A meta-analysis of randomized controlled trials (RCTs) of patients with non-cancer chronic pain indicates that opioids had a significant but small improvement in pain and physical function, though more patients vomited when compared with placebo [5]. When used for other reasons than prescribed, opioids can constitute abuse or dependence [6]. Chronic opioid use can lead to opioid tolerance, which leads a reduced response to the same dosage of opioids that once provided the desired effect [6]. Therefore, individuals with opioid tolerance need to use higher dosages to achieve the same effect, which predisposes them to addiction [6].

The pain alleviating effect of MC is conferred by the therapeutic effect of tetrahydrocannabinol-alpha (THC)—the dominant component of the cannabis extract—and cannabidiol (CBD), a lesser (40%) component of the extract of MC [7]. Cannabis is considered an illicit drug by the US Drug Enforcement Agency (DEA), and it is not approved by the Food and Drug Administration (FDA) [8]. Nevertheless, several US states have policies permitting cannabis use to treat certain medical conditions [9]. A meta-analysis of MC for non-cancer chronic pain reported a significant effect on pain reduction, although its effect was marred with high number needed to treat, and a higher likelihood to harm [10]. More so, compared with placebo, while MC may increase the number of people achieving pain relief, it is associated with an increase in nervous system adverse events [11]. These reports cast doubt on the effectiveness of MC for non-cancer chronic pain. Pain, including back pain, migraine, chronic pain, arthritis, and pain from cancer and surgery, is the most common condition for which MC is prescribed by health providers [6, 8]. When MC is used by patients taking opioids, it does not significantly change the area under the curve (AUC) of opioids or their metabolites, and there is a time delay to maximum serum concentration (Cmax) of opioids [12]. In addition, MC has no significant effect on the pharmacokinetics of opioids [12]. In one study, 35.8% of respondents substituted opioids for MC, with greater substitution among those with comorbidities like pain [13]. Consequently, MC is perceived as an effective remedy for non-cancer chronic pain as well as a potential substitute that may help curb the on-going opioid epidemic [13]. This led to an increasing interest in research on MC, though there is a limited focus on the use of MC for opioid dosage reduction or non-cancer chronic pain. For instance, a systematic review by Whiting et al. included patients with chronic cancer pain and studies that compared CBD to a placebo [14]. Another clinical review by Hill discussed the indications for MC and patient eligibility for MC certification, without an appraisal of MC for non-cancer chronic pain [15]. In addition, a review by Campbell et al. summarized literature on MC use for non-cancer chronic pain [16]. Therefore, in this review, our objective was to assess the effectiveness of MC in reducing opioid dosage or substituting opioids for the treatment of non-cancer chronic pain.

## Methods

### Inclusion criteria

#### Type of studies

Cohort, randomized controlled trials, controlled before-and-after studies, cross-sectional studies, and case reports.

#### Type of participants

Human participants aged 18 years or older who received MC as an adjunct to opioids for the treatment of non-cancer chronic pain. Studies involving cell lines, tissue culture, or animal models were excluded.

#### Type of intervention

Use of MC as an adjunct to opioids in treating non-cancer chronic pain.

#### Type of comparison

Participants who did not receive MC as an adjunct to opioids in treating non-cancer chronic pain.

#### Type of outcome measures

The primary outcome of interest is the reduction of opioid dosage for non-cancer chronic pain treatment.

### Search strategy

A Health Sciences Librarian (AN) developed the search strategy (Additional file 1) for the review and searched PubMed, Web of Science, PsycINFO, and Ovid (Medline). All databases were searched for articles published from inception to October 31, 2019. Two reviewers searched the grey literature using Google and Google Scholar.

### Study selection

**Fig. 1:**
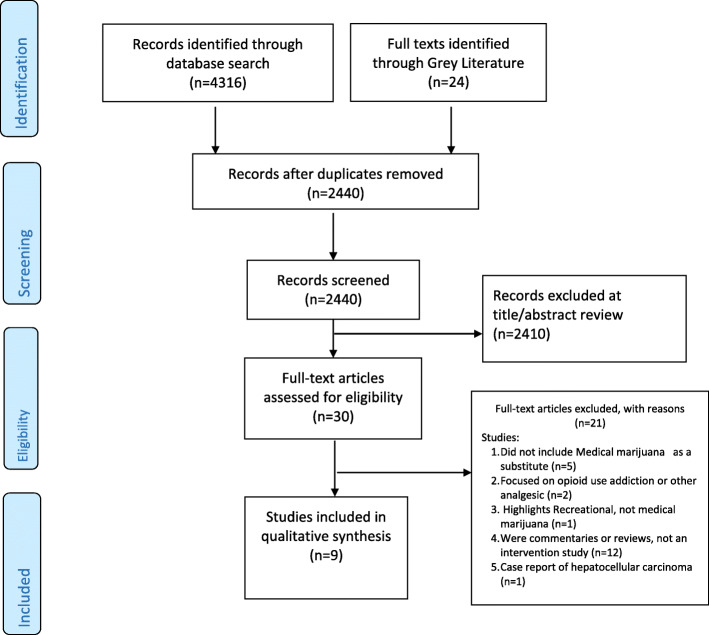
Detailed study selection process

Two reviewers (BO and IA) screened articles against the inclusion criteria, and disagreements regarding study eligibility were resolved by discussion with a third reviewer (JE). Data extraction was done by a reviewer and cross checked by another reviewer. Overall, nine studies were included in the review as shown in the PRISMA diagram (Fig. 1). Studies were eligible for inclusion if they were a cohort study, randomized controlled trials, controlled before-and-after studies, cross- sectional studies, or case reports. The primary outcome of interest is reduction of opioid dosage for non-cancer chronic pain treatment.

### Study quality assessment

Quality assessment of included studies was conducted independently by two reviewers (LK and BO), using the ROBINS-I risk of bias tool for cohort studies and the AXIS tool for cross-sectional studies [17]. Disagreements were resolved by discussion. Cohort studies were assessed for bias related to (1) confounding, (2) selection of participants, (3) classification of interventions, (4) deviations from intended interventions, (5) missing data, (6) measurement of outcomes, and (7) selection of the reported result. Each section of the bias assessment was judged to see if there was a low, moderate, serious, or critical risk of bias. An overall assessment of the risk of bias was made based on the most severe form of risk of bias reported in any of the domains. The cross-sectional studies were assessed for bias in each section of the publication as in Additional file 1: Introduction, Methods, Results, Discussion, and Others. Risk of bias criteria were assessed as “Yes,” “NO,” or “Do not know” (Additional file 1). Given the heterogeneity of included studies, a meta-analysis was not possible. Thus, a qualitative summary of the evidence was conducted.

## Results

The search yielded 4316 articles and 24 reports from the databases and grey literature, respectively. One thousand and nine hundred duplicates were eliminated, leaving 2440 unique studies. Two authors screened the 2440 studies and selected full texts of nine studies that qualified for inclusion (Fig. 1).

### Characteristics of included studies

The search of the four databases yielded 4316 titles, while the grey literature search provided additional 24 research titles. Two thousand, four hundred and forty (2440) titles were remaining after the removal of duplicates; 2410 titles were ineligible and screened out at the abstract stage. Thirty (30) full-text articles were screened, out of which 21 were excluded (Additional file 1).

Nine observational studies involving 7222 participants were included in this review. Included studies (three cohort [18–20], five cross-sectional [21–25], and one case series [26]) were published between 2003 and 2019 in Australia, Canada, and the USA. Although most of the studies did not report the dosage of MC, two reported MC dosage range of 1.5–2000 mg [23, 24]. The participants ranged in age from 34 to 70 years old. See Table 1, Characteristics of included studies, for detailed indications for and the setting of administration of MC.

**Table 1:** Characteristics of included studies

**1**	**Barlowe 2019** [18]	
	Methods	Retrospective cohort study
	Participants	Patients at Dartmouth-Hitchcock Medical Center enrolled in active opioid contracts for painful chronic pancreatitis
	Intervention	35 out of 53 patients were registered with a state therapeutic cannabis program in either New Hampshire or Vermont. Study did not report method of use of medical cannabis (MC).
	Outcomes	Opioid prescription dose was converted into a morphine equivalent dose (MED). Patients registered on the cannabis program showed a decreased mean daily opioid use of 126.6 MED (± 195.6 MED) compared to those who were not enrolled 183.5 MED (± 284.5 MED), *P* = 0.39. Patients enrolled in state therapeutic cannabis programs had decreased mean hospital admissions in the past calendar year as compared to the unenrolled group; *P* = .53 had reduced number of visits to the emergency department in the past year as compared to those enrolled in the active opioid contract (*P* = 0.59) and a fall in mean emergency department visits in the past calendar year as compared to patients not enrolled (*P* = .39). Average daily opioid use in patients at initiation time is 190.34 MED (± 273.3 MED).
**2**	**Boehnke 2016** [21]	
	Methods	Cross-sectional survey through online questionnaires to medical cannabis patient
	Participants	244 medical cannabis patients with CP who patronized a medical cannabis dispensary in Michigan between 2013 and 2015. Survey has 46 questions detailing medical conditions for which MC was used and participants completed the 2011 Fibromyalgia Survey Criteria to stratify level of pain. Study did not report methods of use of MC.
	Intervention	No intervention, however, survey was on participants who were already on medical cannabis
	Outcomes	Patients with lower pain centralization had the largest reductions in opioid use as compared to those who reported higher levels of pain centralization. Mean change in self-reported opioid use was − 64%
**3**	**Campbell 2018** [20]	
	Methods	Cohort study with a 4-year follow-up. Baseline interviews and self-completed surveys were used to get participants’ responses.
	**Participants**	1514 participants, 18 years or older using opioids, recruited across community pharmacies across Australia. Although the questionnaire asked about the methods of use of MC, the study did not report on this.
	Interventions	**None**
	Outcomes	At 4-year follow-up, 24% of participants had used MC for pain. At 3-year and 4-year follow-up waves, 78% and 70% of participants with adjuvant MC usage reported no effects of MC on opioid use, respectively. Also, at 3-year and 4-year follow-up waves, 22% and 30% of participants with adjuvant MC usage reported an occasional or regular reduction of opioids when using MC.
**4**	**Degenhardt 2015** [22]	
	Methods	Community survey of a sample of people previously prescribed opioids for non-cancer chronic pain. Study included 1514 people in Australia to collect data on cannabis use, ICD10- cannabis use disorder, and cannabis use for pain.
	Participants	1514 participants who had previous prescription of medical cannabis. Study did not report on method of use of MC.
	Intervention	No intervention, however, survey was on participants who were already on medical cannabis.
	Outcomes	16% of the cohort used medical cannabis for pain relief on the survey month. Average pain relief was 70%. In contrast, the average reported pain relief they reported from opioid medication was 50%. Those who used medical cannabis were mostly younger, had greater pain severity, were on higher opioid doses, and were more likely to be non-adherent to the prescribed opioid medication. Of those who had used cannabis for pain relief, *n* = 34 felt that cannabis provided 100% pain relief; only four of these reported that their medications gave them 100% pain relief (and among all those using cannabis for pain relief, *n* = 10 reported 100% pain relief from their medications).
**5**	**Lucas 2017** [24]	
	Methods	Cross-sectional survey of registered customers of Tilray a registered producer of medical cannabis.
	Participants	301 participants (53%) used medical cannabis for chronic pain. Methods of MC use include joints (243; 90%), vaporizers (*n* = 234; 86%), oral/edibles (baked goods, butter, tincture, etc.) (207; 76%), and cannabis-infused topical ointments (44;16%).
	Intervention	No intervention; however, survey was on participants who were already on medical cannabis
	Outcomes	73% use medical cannabis for CP; 335 of participants reported substituting opioids with medical cannabis.
**6**	**Lucas 2019** [23]	
	Methods	Cross-sectional survey collected via email from Canadian medical cannabis patients collected information on patterns of use and impact of medical cannabis on use of prescription drugs, tobacco, illicit substances, alcohol, and tobacco.
	Participants	2032 participants, 91% Caucasian, and 62% males. Primary method of use of MC was vaporizer (31.1%), joint (30.4%) oral/edible (16.3%), pipe (11.3%) waterpipe/bong (10.4%), topical (0.3%, juicing (0.2%)
	Intervention	No intervention, however, survey was on participants who were already on medical cannabis.
	Outcomes	Prescription drugs were the most cited substances that cannabis was used to substitute (69.1%). 35.3% of theses prescription medicines was opiates and opioids. Patients cited the following reasons by rank for substitution: a safer alternative, fewer adverse effects, better symptom management, fewer withdrawal symptoms, ability to obtain medical cannabis, and greater social acceptance of cannabis than prescription drugs.
**7**	**Lynch 2003** [26]	
	Methods	Case series of three patients who used small doses of smoked marijuana in combination with an opioid.
	Participants	Patient A: a 47-year-old woman with a 10-year history of chronic progressive multiple sclerosis with significant ambulatory function from joint pain and leg spasticity. Opioid regiment was long acting morphine 75 mg per day, tizanidine 24 mg per day, and Sertraline 150 mg at bedtime. Patient B: 35-year-old HIV positive with painful peripheral neuropathy. Opioid regiment consisted of long-acting morphine 360 mg per day with morphine sulfate 75 mg 4 times daily and gabapentin 2400 mg per day. Patient C: a 44 year-old-man with a 6-year lower back and leg pain following a traumatic fall. Opioid regiment was long acting morphine, 150 mg per day and cyclobenzaprine 10 mg three times per day. Methods of use of MC were smoked marijuana for the three patients.
	Intervention	Patient A: 2–4 puffs of smoked marijuana at bedtime. Morphine regiment decreased. Patient B: 3–4 puffs 3–4 times per day. The morphine regiment decreased over 2 years. Patient C: Several puffs to one joint 4–5 time per day.
	Outcome	Patient A reported improvement in pain. Patient B reported an improvement in pain except during an infection with herpes zoster and discontinued morphine after 2 years. Patient C reported improvement in pain and was able to reduce his dose of morphine.
**8**	**Piper 2017** [25]	
	Methods	Convenient Sampling method for s cross sectional survey
	Participants	1513 participants from a convenient sampling of members of dispensaries of New England, USA, primarily from Maine, Vermont, and Rhode Island. Study did not report method of use of MC.
	Intervention	215 regularly used opioids, 70% use MC for CP reported use of opioids with cannabis.
	Outcomes	76.7% reported a reduction in their opioid use, slightly or a lot since initiating medical cannabis.
**9**	**Vigil 2017** [19]	
	Methods	Quasi-experimental study of 37 habitual opioid users for chronic pain enrolled in the Medical Cannabis Program (MCP) compared to 29 unenrolled patients over 21 months.
	Intervention	No intervention, however, survey was on participants who were already on medical cannabis. Study did not report on methods of use of MC.
	Outcomes	The medical cannabis patients had 5.12 higher odds of reducing daily prescriptions of opioids with improvements in pain reduction, quality of life, social life, and activity levels.

### Quality assessment of included studies

One cohort study [18] had a serious risk of confounding and did not provide enough information to make an overall risk of bias assessment. The other cohort study [19] had a serious risk of bias related to missing data and inadequate measurement of outcomes The third cohort study [20] had a serious risk of bias for confounding and measurement of outcomes, and critical risk of bias related to missing data, with an overall critical risk of bias assessment. See Additional file 1 for the risk of bias assessment of included cohort studies.

A complete assessment of the risk of bias for the five included cross-sectional studies is presented in Additional file 1. One study [21] had no clear study objectives, and three [21, 23, 24] had poor outcome measurement. Also, it was unclear what was used to determine statistical significance or precision estimates for the studies [21, 23, 24]. In two of the studies [21, 24], the research methods were insufficiently described to facilitate possible replication. Two others [23, 24] had funding sources or conflicts of interest that might affect authors’ interpretation of the results; they contributed 30% (2333/7222) of participants in the systematic review.

### MC use and reduction of opioids dosage

Among a cohort of 35 MC users in the cannabis program of New Hampshire or Vermont, USA, there was a reduction in mean daily opioid usage of 126.6 mg, compared to 138.5 mg in those not on the program [18]. In the same population, there was also reduction in mean emergency department visits and hospital admissions from chronic pain in the preceding calendar year [18]. Furthermore, in 37 habitual opioid users for chronic pain enrolled in the medical cannabis program, patients on MC were more likely to reduce daily opioid dosage than those not using MC (83.8% vs. 44.8%) over a 21-month period [19]. A cohort study, with a 4-year follow-up period, reported an occasional or regular reduction of opioid use with MC in 22% and 30% of participants on the 3rd and 4th year follow-up waves, respectively [20]. In a cross-sectional online survey of 1513 members of dispensaries in New England, USA, 76.7% of patients with non-cancer chronic pain using opioids reduced opioid use after starting MC [25]. Similarly, a sample of 244 MC patients with non-cancer chronic pain attending a Michigan MC dispensary reported a 64% reduction in opioid use after starting MC [21], and 18.4% of 2032 Canadian MC patients reported up to a 75% reduction in opioid dosage [23]. In a case series of three patients with non-cancer chronic pain of 6–10 years duration, the use of MC led to 60–100% reduction in the opioid dosage compared to when MC was not used [26]. Among 1514 respondents who used MC for non-cancer chronic pain in Australia, there was an average of 70% pain relief, where 100% meant complete pain relief [22].

### MC use and opioid substitution

Three of the included studies reported an outright substitution of opioids with MC in patients with non-cancer chronic pain [19, 23, 24]. There was opioid substitution with MC in 40.5% of MC users compared to 3.4% in non-users [19]. Amongst MC users in a Canadian MC program, opioid medications accounted for 35.3% (610/1730) of all prescription drug substitutions [23], with 32% (80/251) [24] and 59.3% (362/610) [23] of participants using MC for non-cancer chronic pain reporting an outright stoppage of opioids.

## Discussion

The goal of this review was to assess the use of MC as an adjunct to opioids to reduce opioid dosage in the treatment of non-cancer chronic pain. After screening eligible studies, we found nine studies that reported using MC to reduce opioid dosage for the treatment of non-cancer chronic pain. This review found a much higher reduction in opioid dosage, reduced emergency room visits, and hospital admissions for chronic non-cancer pain by MC users, compared to people with no additional use of MC. There was 64–75% reduction in opioid dosage for MC users and complete stoppage of opioid use for chronic non-cancer pain by 32–59.3% of MC users, when compared to patients without additional use of MC.

The strength of the evidence is the adoption of a rigorous standard approach to the review, based on the PRISMA checklist, the inclusion of publications from four databases, and the independent screening of study eligibility. Given the dearth of empirical studies about MC versus opioids for the treatment of non-cancer chronic pain, it is important that readers have information on the full range of currently available evidence. Thus, this review relaxed inclusion criteria allowing for the inclusion of observational studies, including case reports. Though findings from the nine included studies suggest that medical cannabis may be used as an adjunct with opioids to reduce opioid dosage when treating non-cancer chronic pain, it is limited by the fact that it is derived from self-reports of reduction of opioid dosage as well as the fact that most included studies did not report the MC dosage that led to reduction of opioid dosage. More so, a study that reported a 22–30% reduction of opioid medication use, when MC is used as an adjunct, equally stated that 70–78% of participants reported no influence of MC on the use of opioids [20]. The wide range of MC dosage (1.5–2000 mg) reported by two cross-sectional studies suggests the difficulty in arriving at a standardized MC dosage for patients with non-cancer chronic pain. Furthermore, included cohort studies were assessed as having serious or critical risk of bias overall. The lack of measures previously published to assess study outcomes, unclear precision estimates, and insufficiently described methods for these studies underscore the need for caution in interpretation of findings.

The availability of, and access to, MC in states with MC laws implies that patients with non-cancer chronic pain who do not obtain relief with common medications might consider an MC prescription. Patient caregivers might suggest trialing MC to relieve pain or avoid the undesirable side effects of long-term opioid use, including dependence and addiction. Therefore, more Americans are likely to turn to MC especially with an estimated 50 million living with non-cancer chronic pain [3].

While this review indicates the likelihood of reducing opioid dosage when used in combination with MC, there are shortcomings. One challenge is not knowing the optimal MC dosage to achieve opioid dosage reduction. Further, studies are needed to gradually increase MC dosage titrated against a reduction in opioid dosage until an optimal pain relief effect is attained. A more notable concern is the fact that none of the included studies discussed potential adverse effects of using MC as an adjunct to opioids. It is known that THC, the active ingredient of MC, reduces gastrointestinal motility, drug absorption, and metabolism [15, 22], resulting in reduced opioid absorption, and lowers the potential for addiction. MC used in combination with opioids in the treatment of non-cancer chronic pain may equally have yet unknown health consequences. Thus, there is an urgent need for well-planned research studies to validate current evidence in the scientific literature. Large-scale and experimental studies are needed to better understand MC’s use as an adjunct to opioids for treating non-cancer chronic pain. Irrespective of the route of administration used, the different pharmacokinetic properties of medical cannabis dictate that standardized cannabis composition and packages should be used to allow for comparison of research findings.

In states where MC is legal, future research should assess the effects of long-term MC use on opioid addiction and opioid-related deaths. Additionally, there is a need to assess the optimal/standardized MC dosage to achieve a reduction in opioid dosage and what routes of MC administration would most reduce opioid dosage the fastest. Researchers must also assess the long-term health and wellness consequences of reduced gastrointestinal motility reported to be beneficial to reduce opioid dependence and opioid-related mortality.

## Conclusion

Given the current opioid epidemic in the USA and medical cannabis’s recognized analgesic properties, MC could serve as a viable option to achieve opioid dosage reduction in managing non-cancer chronic pain. Unfortunately, the evidence from this review cannot be relied upon to promote MC as an adjunct to opioids in treating non-cancer chronic pain. The nine available studies included in this review suggest that cannabis was effective as an adjunct to opioid in reducing the dosage of opioids in study participants. However, the design of included studies provides a limited basis on which to make a rational, evidence-based recommendation. As the USA grapples with the opioid abuse epidemic and searches for less addictive alternatives, experimental studies are urgently needed to assess the effects of cannabis on non-cancer chronic pain as well as its potential to reduce the need for opioids. If cannabis is found to be effective in reducing non-cancer chronic pain, it could serve as a viable substitute for prescription opioids, thus mitigating the opioid epidemic.

## Supplementary information

**Additional file 1.** Appendices.

## Data Availability

The systematic review included published studies that are readily available to the public.

## Abbreviations

AUC: Area under the curve
CBD: Cannabidiol
Cmax: Maximum serum concentration
DEA: U.S. Drug Enforcement Agency
FDA: Food and Drug Administration
MC: Medical cannabis
THC: Tetrahydrocannabinol alpha
