# Supplementary material for: Medical cannabis for the reduction of opioid dosage in the treatment of non-cancer chronic pain: a systematic review
Source: Syst Rev. 2020 Jul 28;9:167. doi: 10.1186/s13643-020-01425-3 (PMC7388229; doi:10.1186/s13643-020-01425-3)
Supplement: Supplementary file 1 — Additional file 1. Appendices. [file 13643_2020_1425_MOESM1_ESM.docx]

**APPENDICES**

**Appendix 1: Search strategy**

**MEDLINE PUBMED**

("Cannabis"[Mesh] OR "cannabis"[all fields] OR "Marijuana smoking"[Mesh] OR "marijuana smoking"[all fields] OR "Marijuana Abuse"[Mesh] OR "marijuana abuse"[all fields] OR "cannabis abuse"[all fields] OR "Marijuana Use"[Mesh] OR "Marijuana Use"[all fields] OR "cannabis use"[all fields] OR "Medical Marijuana"[Mesh] OR "medical cannabis"[tiab] OR "medical marijuana"[tiab]) AND ("Analgesics, Opioid"[Mesh] OR "opioid epidemic"[all fields] OR "opioids"[all fields] OR "Opioid-Related Disorders"[Mesh] OR "opioid overdose"[All Fields] OR "opioid abuse"[All Fields] OR "opioid use"[all fields] OR "opioid addiction"[all fields] OR "prescription opioid"[all fields] OR "opioid dependence"[all fields] OR "opioid therapy"[all fields] OR "opioid withdrawal"[all fields])

**MEDLINE OVID**

exp Cannabis/ OR cannabis.mp. OR exp marijuana smoking / OR marijuana smoking.mp. OR exp marijuana abuse/ OR marijuana abuse.mp. OR cannabis smoking.mp. OR exp medical marijuana/ OR medical marijuana.mp. OR medical cannabis.mp. AND

exp Analgesics, Opioid/ OR opioid epidemic.mp. OR opioids.mp. OR exp opioid-related disorders/ OR opioid overdose.mp. OR opioid abuse.mp. OR opioid addiction.mp. OR prescription opioid.mp. OR opioid dependence.mp. OR opioid therapy.mp. OR opioid withdrawal.mp.

**WEB OF SCIENCE**

TS=Cannabis OR TS=marijuana OR TS=marijuana smoking OR TS=marijuana abuse OR TS= marijuana use OR TS=cannabis smoking OR TS=cannabis abuse OR TS=cannabis use OR TS=medical marijuana OR TS=medical cannabis AND TS=analgesics, opioid OR TS=opioid epidemic OR TS=opioids OR TS=opioid-related disorders OR TS=opioid overdose OR TS=opioid abuse OR TS=opioid use OR TS= opioid addiction OR TS=prescription opioid OR TS=opioid dependence OR TS=opioid therapy OR TS=opioid withdrawal

**PsycINFO**

DE Cannabis OR DE Marijuana OR TX “marijuana smoking” OR TX “marijuana abuse” OR DE Marijuana Usage OR TX “marijuana use” OR TX “cannabis smoking” OR TX “cannabis abuse” OR TX “cannabis use” OR TX “medical marijuana OR TX “medical cannabis” AND (DE "Substance Use Disorder" AND DE "Opiates") OR DE "Opiates" OR TX "opioids" OR TX “opioid epidemic OR TX “opioids OR TX “opioid-related disorders” OR TX “opioid overdose” OR TX “opioid abuse” OR TX “opioid use” OR TX “ opioid addiction” OR TX “prescription opioid” OR TX “opioid dependence” OR TX “opioid therapy” OR TX “opioid withdrawal”

**Appendix 2: Table of excluded studies**

| **Article-ID** | **Reason for Exclusion** |
| --- | --- |
| Bacchuber, 2014 | Ecological study that does not address medical marijuana as a substitute for opioids. |
| Bagcchi, 2014 | Study is a review focused on legal aspects of medical marijuana |
| Carter, 2011 | Study is not an original scientific article |
| Carter, 2015 | Study does not include human subjects |
| Choo, 2016 | Study is not an original scientific article |
| Cichewicz, 2004 | Study focuses on pharmaceutical properties of opioids |
| Collen, 2012 | Study is a commentary based on other scientific articles |
| Elikottil, 2009 | Study is a review |
| Hayes, 2014 | Study is not an original scientific article |
| Hsu, 2016 | Report article, not an original scientific study |
| Hurd, 2016 | Study is a report, not an original scientific study |
| Kaskie, 2017 | Study is a review |
| Livingston, 2017 | Study does not address medical marijuana as a substitute for opioids |
| Lucas, 2012 | Study is a review |
| Lucas, 2017 | Study is a review |
| McCarty, 2018 | Commentary article, not an original study |
| Meng, 2016 | Case report on hepatocellular carcinoma |
| Peloquin, 2011 | Journal issue focused on heroin addiction, not the substitution of opioids for medical marijuana |
| Perron, 2015 | Study does not examine the substitution of opioid for medical marijuana |
| Shi, 2017 | Study does not examine the substitution of opioid for medical marijuana |
| Wilson, 2017 | Study does not examine the substitution of opioid for medical marijuana |

**Appendix 3: ROBINS-I risk of bias of included cohort studies**

| Risk of Bias Criteria | Study ID | | |
| --- | --- | --- | --- |
|  | Vigil et al. 2017 | Campbell et al. 2018 | Barlowe et al. 2019 |
| Bias due to Confounding | Moderate | Serious | Serious |
| Bias in selection of participants into the study | Low | Low | Low |
| Bias in classification of interventions | Low | Low | Moderate |
| Bias due to deviations from intended interventions | No information | Low | No information |
| Bias due to missing data | Serious | Critical | No information |
| Bias in measurement of outcomes | Serious | Serious | Moderate |
| Bias in selection of the reported result | Low | Low | Low |
| Overall bias | Serious | Critical | No information |

**Appendix 4: AXIS risk of bias of included cross-sectional studies**

| Risk of Bisk Criteria | | Study ID | | | | |
| --- | --- | --- | --- | --- | --- | --- |
|  |  | Borhenke et al. 2016 | Degenhardt et al. 2015 | Lucas et al. 2017 | Lucas et al. 2019 | Piper et al. 2017 |
| Introduction | Were the aims/objectives of the study clear? | No | Yes | Yes | Yes | Yes |
| Methods | Was the study design appropriate for the stated aim(s)? | DNK | Yes | Yes | Yes | Yes |
|  | Was the sample size justified? | DNK | No | DNK | Yes | Yes |
|  | Was the target/reference population clearly defined? (Is it clear who the research was  about?) | Yes | Yes | Yes | Yes | Yes |
|  | Was the sample frame taken from an appropriate population base so that it closely represented the target/reference population under investigation? | Yes | Yes | Yes | Yes | Yes |
|  | Was the selection process likely to select subjects/participants that were representative of the target/reference population under investigation? | Yes | Yes | Yes | Yes | Yes |
|  | Were measures undertaken to address and categorize non-responders? | Yes | DNK | Yes | DNK | Yes |
|  | Were the risk factor and outcome variables measured appropriate to the aims of the  study? | DNK | Yes | Yes | Yes | Yes |
|  | Were the risk factor and outcome variables measured correctly using instruments/measurements that had been trialled, piloted or published previously? | No | Yes | No | No | No |
|  | Is it clear what was used to determined statistical significance and/or precision estimates? (eg, p values, CIs) | No | Yes | No | No | Yes |
|  | Were the methods (including statistical methods) sufficiently described to enable them  to be repeated? | No | Yes | No | Yes | Yes |
| Results | Were the basic data adequately described? | Yes | No | Yes | Yes | Yes |
|  | Does the response rate raise concerns about non-response bias? | No | DNK | No | No | No |
|  | If appropriate, was information about non-responders described? | No | No | Yes | No | No;  N/A |
|  | Were the results internally consistent? | Yes | Yes | Yes | Yes | Yes |
|  | Were the results for the analyses described in the methods, presented? | DNK | Yes | Yes | Yes | Yes |
| Discussion | Were the authors’ discussions and conclusions justified by the results? | Yes | Yes | Yes | Yes | Yes |
|  | Were the limitations of the study discussed? | Yes | Yes | Yes | Yes | Yes |
| Others | Were there any funding sources or conflicts of interest that may affect the authors’  interpretation of the results? | DNK | No | Yes | Yes | No |
|  | Was ethical approval or consent of participants attained? | Yes | Yes | Yes | Yes | Yes |
